# Complexities and complications of extreme obesity

**DOI:** 10.4322/acr.2021.402

**Published:** 2022-10-05

**Authors:** Haval Ali, Udit Naik, Michelle McDonald, Mohammad Almosa, Karen Horn, Alexis Staines, Louis Maximilian Buja

**Affiliations:** 1 University of Texas Health Science Center at Houston (UTHealth Houston), McGovern Medical School, Department of Pathology and Laboratory Medicine, Houston, TX, USA

**Keywords:** Obesity, Morbid, Heart Failure, Pulmonary Embolism

## Abstract

Obesity is a common chronic disorder and has detrimental long-term consequences if left untreated. Herein, we report a case of a young lady who suffered from morbid obesity and many of its consequences, and we present a literature review of these complications. While the cause of obesity is multifactorial, the genetic component is particularly important in the pathophysiology of marked obesity. Resistance to Leptin is considered one of the main causes of obesity. There is a unique relationship between polycystic ovary syndrome and obesity, as observed in our case. Obesity is associated with cardiovascular and lung diseases such as heart failure, thromboembolic disease, sleep apnea, and pulmonary hypertension. Our patient had cardiomegaly (730 gm) with eccentric hypertrophy of left and right ventricles. The coronary arteries and aorta were free of atherosclerosis, which is a surprising finding that relates to the mysterious phenomenon of obesity paradox. The terminal event in our young woman was multiple segmental and subsegmental pulmonary arterial thrombi/thromboemboli superimposed on chronic cardiopulmonary stress due to massive obesity.

## INTRODUCTION

Obesity can be defined as increased body fat mass due to excessive calorie surplus. For adults, the World Health Organization (WHO) uses the body mass index (BMI) to define and classify obesity, even though BMI does not measure the body fat directly.^1^ BMI is calculated by dividing the body weight, using kilograms as a measuring unit, by the square of the body height, using meters as a measuring unit.^2^ The WHO considers an adult with a BMI greater than or equal to 30 kg/m^2^ to be obese. Obesity is subcategorized into three classes. A BMI of more than or equal to 40 kg/m^2^ (or at least 35 kg/m^2^ with comorbid conditions) is considered class III or extreme obesity (AKA morbid obesity, severe obesity).^3^^,^ 4 According to the most recent data by the National Health and Nutrition Examination Survey (NHANES), it is estimated that 73.6% of the United States adult population have a BMI above the normal limit (above 25 kg\m2), out of which 33.5% are classified as obese, and 9.0% are classified as severely obese.^5^

Obesity is considered a chronic disease and requires extended medical attention. It is associated with various chronic conditions that affect multiple body systems, such as cardiovascular, endocrine, respiratory, gastrointestinal, hepatobiliary, and immune systems. Obesity increases the risk of developing diabetes mellitus, hypertension, coronary heart disease, heart failure, sleep apnea, non-alcoholic fatty liver disease, osteoarthritis, and depression.^6^^-^ 11 It is reported that the mortality rate of obese individuals is 50% higher compared to individuals with normal BMI.^4^

Herein we report the case of a young woman who, at age 33, met the criteria for class III extreme obesity. After a 3-week history of intermittent shortness of breath, she developed a sudden cardiovascular collapse and demise. Autopsy revealed pathological processes involving multiple organs. We review the complexities and complications of extreme obesity demonstrated by this case.

## CASE REPORT

The subject was a 33-year-old massively obese female who was brought to our hospital by the Emergency Medical Service after experiencing a collapse at home. Four years prior to this event, she had been seen in the Emergency Department of our hospital with complaints of shortness of breath and heavy menstrual bleeding. Vital signs included blood pressure recordings in the range of 160-167/67-80 mmHg. Blood hemoglobin and hematocrit were 5.9 grams per deciliter (g/dl) and 21.3 percent (%), respectively. The woman was given a bolus of medroxyprogesterone and transfused with 2 units of packed erythrocytes which resulted in a rise of the hemoglobin to 7.5g/dl. She was started on oral iron medication and discharged. She was not seen again in our health care system until she was brought to our Emergency Medical Department in extremis.

A history given by the woman’s mother revealed that the woman had been complaining of shortness of breath for the past 3 weeks. The mother stated that she had a history of asthma and anemia and that she had recently been seen by a primary care physician, who had treated her with a diuretic due to bilateral leg swelling. The mother reported that subsequently the woman became acutely distressed one morning and complained that she was “hurting all over”. She was on her way to her primary care physician when she was found unresponsive and pulseless. Cardiopulmonary resuscitation (CPR) was initiated by bystanders, Emergency Assistance was not called until 10 minutes after. When the emergency medical service (EMS) arrived, she was found to have a “non-shockable rhythm.” Thirteen rounds of cardiopulmonary resuscitation (CPR) were performed, and 8 doses of epinephrine were given. The EMS was unable to intubate her given her clinched jaw, so a nasopharyngeal airway was placed instead. On arrival at the emergency room, CPR had been performed for approximately an hour. CPR was continued. The clinical team was unable to intubate her given her body habitus; however, laryngeal mask airway (LMA) was placed successfully. Despite ongoing CPR, the woman exhibited pulseless electrical activity (PEA), which was resistant to resuscitative efforts. She was pronounced dead in the Emergency Room. Permission for an unrestricted autopsy was obtained.

## AUTOPSY FINDINGS

The external examination of the decedent showed an obese female weighing 227.2 kilograms with body mass index (BMI) of 86 kg/m^2^. Waist circumference was increased with increased ratio of waist to hips. Fat was mainly accumulated around the abdomen, chest, neck, and upper extremities, which was consistent with apple-shaped/central obesity. There was dark, coarse hair growth on the chin, submental, submandibular, and upper neck areas. The legs were symmetric with equal thigh and calf circumferences and without discoloration or edema. The external genitalia were those of an adult female and without virilization.

**Figure 1:**
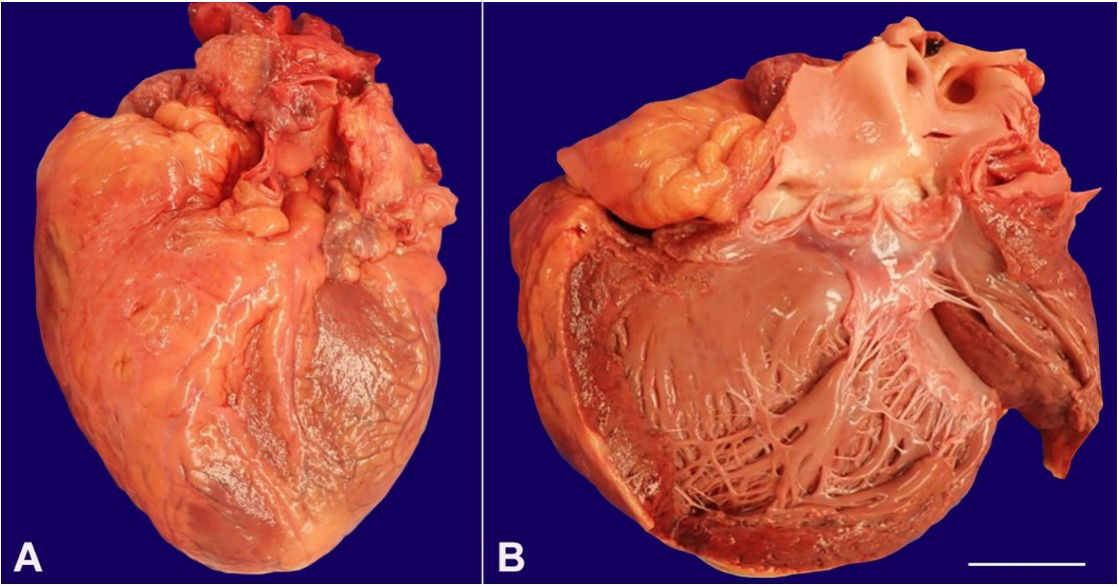
Gross view of the heart. **A –** Heart shows enlarged right and left ventricles and weighs 730 grams; **B –** Opened left ventricle has a rounded shape indicative of eccentric hypertrophy (scale bar = 5 centimeters).

**Figure 2:**
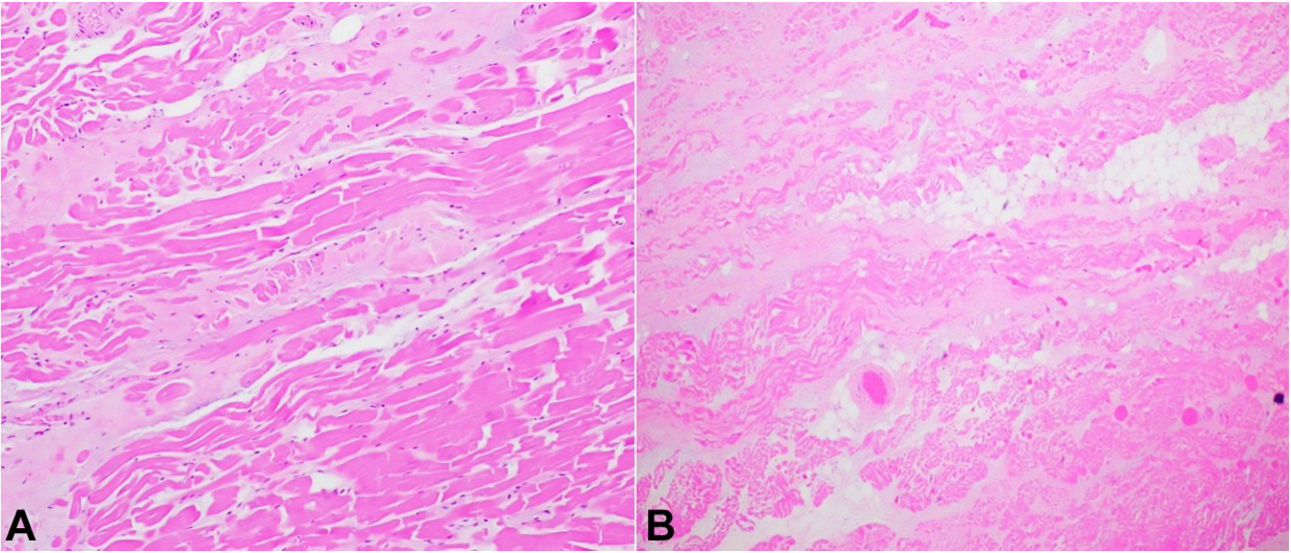
Photomicrographs of the heart. **A –** Myocardium of the right ventricular papillary muscle exhibits interstitial fibrosis (H&E, x 100); **B –** Right atrial myocardium has interstitial fibrosis (H&E, x40).

**Figure 3:**
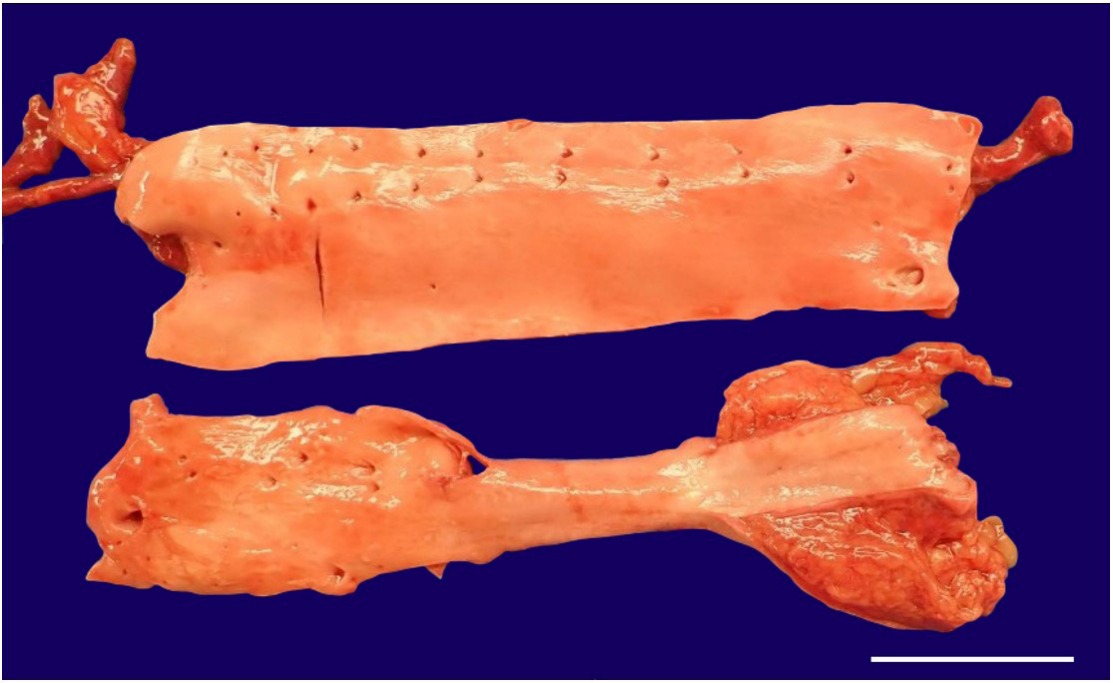
Gross view of the Aorta completely free of atherosclerotic lesions (scale bar = 5 centimeters).

**Figure 4:**
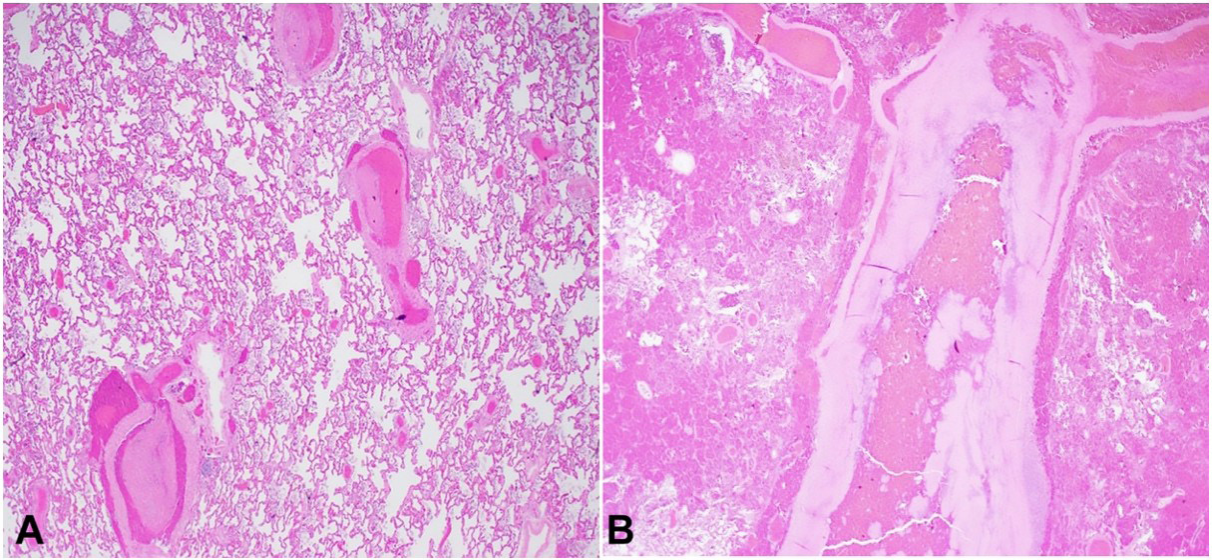
Photomicrographs of the lung. **A –** Subsegmental small pulmonary arteries are occluded by thrombi (H&E, x20); **B –** Segmental branch pulmonary artery is obstructed by a thrombus (Hematoxylin and eosin stain, x20).

**Figure 5:**
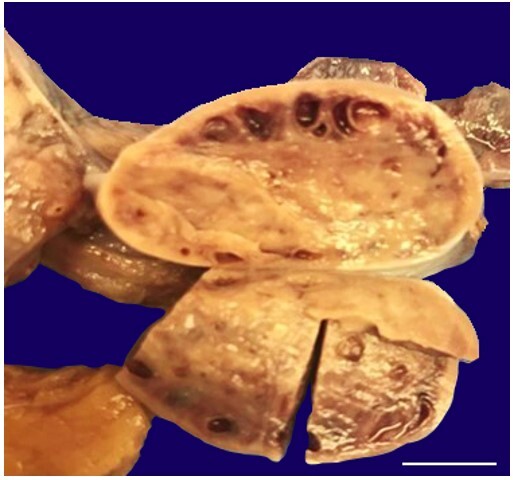
The ovary has multiple cortical cysts. (scale bar = 3.5 centimeters).

The internal examination revealed that the subcutaneous fat layer of the abdominal wall was 12 centimeters thick. Examination of the internal organs revealed multiple findings of note (Figures 1-5).

There was cardiomegaly (730-gram heart) with an increased amount of epicardial fat. The coronary artery system displayed patent ostia and a left dominant distribution. The coronary arteries were widely patent. The left and right ventricles were hypertrophied and dilated (Figure 1). No pallor, softening, hemorrhage or fibrosis were identified.

Microscopically, the cardiomyocytes were hypertrophied with enlarged nuclei and abundant eosinophilic cytoplasm. There was interstitial fibrosis and mild fatty infiltration in the papillary muscles and atria (Figure 2).

The aorta and its major branches were free of atherosclerosis or other abnormalities, both macroscopically and microscopically (Figure 3). The venae cavae and their major tributaries had their usual distribution and were free of thrombi.

The weight of the lungs was significantly increased (1100 grams left, 1200 grams right). The cut surfaces of the parenchyma exuded a moderate amount of sanguineous fluid. Examination of the vasculature revealed diffuse scattered thrombi/emboli in segmental and subsegmental muscular pulmonary arteries. These findings were also identified in the microscopic examination (Figure 4).

There was hepatosplenomegaly with no visible or palpable fibrosis. The microscopic examination of the liver exposed diffused mild hepatic steatosis with mild degree of fibrosis around the portal triad. The gallbladder had multiple small black stones.

The ovaries were not enlarged (each 3.5 × 2 × 1 cm), but the parenchyma of both ovaries exhibited multiple cystic lesions in the cortical areas (Figure 5).

A calcified endometrial polyp was identified in the lower uterine segment. The microscopic examination corresponded with the macroscopic findings.

The significance of these findings is summarized in Table 1.

**Table 1:** Autopsy findings in a massively obese 33-year-old woman

Category	Findings	Findings’ significance
**Clinical history**		
History of anemia due to oligo-ovulation and menorrhagia	Morbid / severe central obesity	Polycystic ovary syndrome^12^
	Hirsutism	
**External**		
Weight (BMI)	227.2 kg, BMI: 86 kg/m^2^	Class 3 obesity (Morbid/severe)^3^^,^ 4
Features of note	Thick, dark hair especially on the neck (hirsutism), increased waist circumference and ratio of waist to hips (upper body obesity)	Polycystic ovary syndrome, obesity and metabolic syndrome^12^
**Cardiovascular System**		
Heart weight	Cardiomegaly 730 g*	Heart failure^13^^-^ 17
Heart chambers and walls	Dilated and thickened left ventricle; Fibrosis of the right atrium and right papillary muscles	Volume overload and pulmonary hypertension.
		Obesity-induced fibrosis. Obstructive sleep apnea^13^^-^ 17
		Obesity cardiomyopathy^17^
Coronary arteries	Patent	Obesity paradox^18^^-^ 21
Aorta	Normal/without pathological alteration	Obesity paradox^18^^-^ 21
**Respiratory System**		
Lung weight	Weight increased; L 1100 g, R 1200 g**	Pulmonary edema
Vasculature	Microscopically: thromboembolic lesions in the small & intermediate pulmonary vessels	Acute phase of thromboembolic disease^22^^-^ 28
**Hepatobiliary System**		
Liver weight	Hepatomegaly; 2300 g**	Congestive heart failure
Liver microscopic findings	Fatty liver with low grade fibrosis of the portal triad	Nonalcoholic fatty liver disease (NAFLD) and Metabolic syndrome^10^
Gall Bladder	Gallstones	Cholelithiasis due to obesity^8^
**Genitourinary System**		
Kidney weights	Weight increased; L 210 g	Consistent with hyperfiltration due to obesity^9^
	R 210 g.**	
	Increased glomerular size	
**Reticuloendothelial System**		
Spleen weight	Splenomegaly; 450 g**	Early portal hypertension or right-side heart failure, or increased growth factor due to obesity^11^
**Endocrine System**		
Body fat	Increased body fat and obesity	Leptin resistance, insulin resistance^8^^,^ 9 and polycystic ovary syndrome^12^
**Central Nervous System**	No pathological alteration	
**Reproductive**		
Uterus	Endometrial polyp	Polycystic ovary syndrome^12^
Ovaries	Multiple cysts on both ovaries	Polycystic ovary syndrome^12^

* Expected heart normal weight: 148 to 296 g;^17^

** Expected other organs normal weight: Liver, 603 to 1767 g; spleen, less than 230 g; right lung, 101 to 589 g; left lung, 105 to 515 g; right kidney, 38 to 174 g; and left kidney, 35 to 192 g.^29^

## DISCUSSION

Obesity is characterized by a disproportionate body weight for height with an excessive accumulation of adipose tissue and is often accompanied by a state of mild, chronic, systemic inflammation.^7^ Obesity leads to the development of metabolic syndrome, type II diabetes mellitus, cardiovascular diseases, some forms of cancer, and other pathological conditions.^7^ There is a J-shaped relationship between BMI and morbidity and mortality, with increased health risk associated with extreme leanness and obesity.^7^ This risk is highest for Class III extreme obesity, as manifested by our subject. Our autopsy investigation documented a constellation of multiple abnormalities that contribute to the high morbidity and mortality of extreme obesity.

The effect of the environment and the psychosocial status (such as ready availability of calorie-dense food and a sedentary lifestyle) have contributed to the rise in obesity prevalence in recent years.^6^ However, like most chronic diseases, the mechanism of developing obesity can be explained by the multifactorial theory of disease causation, which includes genetic, environmental, and psychosocial factors.^7^ The interplay between these factors affects the fat deposition in the body. This is achieved through several physiological pathways that affect the appetite and energy expenditure.^7^ It is found that overexpression of certain genes can predispose their bearers to obesity.^7^ Overexpression of the fat mass and obesity-associated gene (FTO) is one of the well-established examples of these genes. FTO gene influences body weight by regulating appetite, thermogenesis, adipocyte browning, and epigenetic mechanisms related to obesity.^7^ Other well-known monogenic conditions causing obesity include Melanocortin 4 MC4 receptor deficiency, pro-opio-melanocortin (POMC) mutations, and mutations of genes encoding leptin and its receptor (Lepr). These conditions are rare single-gene defects that cause severe obesity in children. Abnormalities in the FTO gene are assumed as a cause of morbid obesity in the subject of this case report, given her youthful age and long history of morbid obesity; however, due to her limited medical history and lack of genetic testing, the accuracy of such assumption cannot be validated.

Disturbance of the neuroendocrine system is also implicated in the pathophysiology of developing obesity. Adipose tissue is considered as part of the endocrine system due to its ability to secrete many hormones, and signaling molecules termed adipokines.^30^^,^ 31 Leptin is an adipokine hormone produced and secreted by adipose tissue, that plays a role in food intake and energy balance regulation.^30^^,^ 31 Leptin has a positive correlation with body adipose depots.^30^^,^ 31 Through a negative feedback mechanism, leptin binds leptin receptors in the hypothalamus, and triggers a response to reduce feeding and increase energy expenditure. On the other hand, when the level of leptin decreases, the hypothalamus triggers a cascade of responses to counteract the energy deficit by increasing hunger and promoting energy-sparing. Paradoxically, in obese individuals, this inhibitory mechanism of leptin is disrupted, and leptin receptors in the hypothalamus do not respond to leptin despite its elevated level. This phenomenon is called leptin resistance, and it is considered the hallmark of obesity.^30^^,^ 31

No study about obesity would be complete without mentioning the close link between obesity and polycystic ovarian syndrome (PCOS). PCOS is a common female condition characterized by abnormal reproductive, hyperandrogenic, and metabolic features.^12^ PCOS is considered to be the most common endocrine and metabolic disorders in women of reproductive age. The prevalence of PCOS in premenopausal women is estimated to be 6% to 20%, depending on the criteria used to define the syndrome.^12^ PCOS ensues when ovarian follicles fail to ovulate and create corpora lutea in the ovaries. Subsequently, due to the lack of progesterone surge, higher pulse frequency of the gonadotropin-releasing hormone (GnRH) ensues, causing the level of the luteinizing hormone (LH) to exceed the level of the follicle stimulating hormone (FSH). LH stimulates androgen synthesis in theca cells which culminates in hirsutism and increases insulin resistance (through increasing visceral adiposity).^12^ It is worth mentioning that insulin resistance has a positive feedback effect on androgen.^12^ This phenomenon raises the question of whether polycystic ovarian syndrome arises because of obesity, or vice versa. It is currently unknown what the first trigger is, but there is general consensus in the scientific community that insulin resistance and excessive androgen are found together in patients with PCOS. Eventually, the elevated androgen level, in combination with low level of FSH, prevents ovarian follicle maturation and reduces the likelihood of successful ovulation. This leads to formation of cysts in/on the ovaries, which were evident findings in our subject.^12^ Prolonged endometrial exposure to unopposed estrogen, due to ovarian dysfunction and hormonal imbalance, leads to thickening of the endometrium and increases the risk for developing endometrial polyps and cancer.^12^ This results in oligomenorrhea and menorrhagia, which might explain why the deceased suffered from anemia.^12^

Obesity has a plethora of adverse effects on hemodynamics, structure, and function of the cardiovascular system. In particular, accumulation of visceral fat, rather than subcutaneous fat, is associated with increased cardiometabolic risk.^7^^,^ 13 ^-^ 17 The heart in obese subjects is subject to increased hemodynamic stress and also undergoes disturbance in metabolism leading to excess lipid accumulation in the myocardium, i.e., fatty heart.^16^ The result is a distinctive form of heart disease designated as obesity heart disease or obesity cardiomyopathy.^17^ The heart in our case weighed 730 gm, exhibited biventricular hypertrophy and dilatation (eccentric hypertrophy), and had microscopic areas of fibrosis indicative of pathological remodeling. Although the range of normal heart weights in obese subjects is debated, any heart weight over 400 gm in women and 500 gm in men can be considered to be abnormal.^29^^,^ 32 ^-^ 34 Our case qualifies as a case of obesity cardiomyopathy.^17^

Obesity causes the heart to circulate blood through relatively low resistance but large amounts of adipose tissue, which leads to hyperdynamic circulation, increased blood volume and cardiac output. The increase in blood volume increases the preload to the right and left ventricles, resulting in increased wall tension and dilatation of both chambers. When these alterations are sustained, cardiac remodeling ensues. This remodeling over time becomes maladaptive and detrimental.^13^^-^ 15 ^,^ 17 Obesity is also associated with elevated blood pressure, and it is reported that the probability of obese patients having hypertension is higher than the general population. The combination of hypertension and maladaptive remodeling results in left and right ventricle hypertrophy.^13^^-^ 15 On the other hand, obstructive sleep apnea, which is also highly prevalent among obese individuals, causes hypoxia-induced vasoconstriction in the lungs and pulmonary hypertension.^13^^-^ 15 ^,^ 17 This elevation in the afterload pressure is another insult to the right ventricle that leads to right ventricular hypertrophy. Eventually, the dilated and hypertrophied ventricles suffer contractile dysfunction that results in heart failure.^13^^-^ 15 ^,^ 17

While epidemiological studies show a strong linkage of obesity with coronary artery disease, post-mortem studies have found considerable variability in the presence and severity of coronary artery disease in young obese individuals.^35^^,^ 36 The pathology studies are consistent with morbid obesity exerting a protective effect against developing vascular atherosclerosis.^33^^,^ 34 This phenomenon is called “obesity paradox.” It is reported that there is a highly significant inverse correlation between body mass index (BMI) and atherosclerosis of the aortas of morbidly obese decedents (BMI >40 kg/m^2^).^18^ The reasons behind such unexpected yet interesting findings are still under investigation.^19^^-^ 21

Multiple thrombotic lesions in segmental and subsegmental muscular pulmonary arteries may develop as primary in situ thrombi in the setting of pulmonary hypertension.^22^^-^ 25 Absence of evidence of deep vein thrombosis favors in situ thrombosis versus thromboembolic origin. Although sudden death is more often related to larger pulmonary thromboemboli, multiple small thrombi in the setting of pulmonary hypertension can produce sudden arrhythmic death.^25^ Restrictive lung pathophysiology and obstructive sleep apnea likely were predisposing factors in our extremely obese patient.^13^^-^ 15 ^,^ 17 Over time recurrence could give rise to thrombotic arteriopathy and chronic thromboembolic pulmonary hypertension.^26^^-^ 28 Being overweight or obese is the most prevalent risk factor for sudden-unexpected death, which has been documented in 75% of cases.^25^ Our autopsy investigation has documented the cardiopulmonary pathology that can produce pathophysiology resulting in sudden death of the extremely obese individual.
